# Using patient preference to inform ritlecitinib dose selection for alopecia areata treatment

**DOI:** 10.1111/1346-8138.17628

**Published:** 2025-01-15

**Authors:** Brett Hauber, Chiara Whichello, Jonathan Mauer, Ernest Law, Myrto Trapali, Edward Whalen, Dalia Wajsbrot, Nicolas Krucien, Tommi Tervonen, Samuel H. Zwillich, Robert Wolk

**Affiliations:** ^1^ Pfizer Inc. New York New York USA; ^2^ Evidera London UK; ^3^ Evidera Zurich Switzerland; ^4^ Pfizer Groton Connecticut USA

**Keywords:** alopecia areata, JAK inhibitors, JAK3, risk assessment, tyrosine kinase inhibitors

## Abstract

Ritlecitinib is an oral Janus kinase 3/tyrosine kinase expressed in hepatocellular carcinoma (JAK3/TEC) family kinase inhibitor approved for the treatment of severe alopecia areata (AA). Benefit–risk profiles of two doses of ritlecitinib (50 mg vs 30 mg once daily) were evaluated by integrating patient preferences and clinical efficacy and safety estimates for ritlecitinib. A discrete‐choice experiment (DCE) was utilized to elicit preferences for benefit and safety attributes of systemic AA treatments. Benefits included probabilities of ≥80% scalp hair coverage and achieving moderate to normal eyebrows and eyelashes. Potential risks included 3‐year probabilities of serious infection, cancer, and blood clots. Preference estimates were used to calculate the maximum acceptable risk (MAR) that patients would accept for expected increases in benefit from choosing a higher ritlecitinib dose over a lower dose. Ritlecitinib benefits were calculated from the ALLEGRO‐2b/3 clinical trial. MARs were calculated separately for each risk and jointly for all possible combinations. Adults (*n* = 201) with physician‐confirmed ≥50% scalp hair loss from AA participated. To achieve expected increases in the probabilities of ≥80% scalp hair coverage or moderate to normal eyebrows and eyelashes when choosing 50 mg over 30 mg of ritlecitinib, patients would be willing to accept increases in each 3‐year risk up to a mean of 3.88 absolute percentage points (95% confidence interval [CI], 2.86–4.90) for serious infection, 1.63 (95% CI, 1.08–2.18) for cancer, and 5.30 (95% CI, 3.60–7.00) for blood clots. These results, combined with the estimated differences in risks between the two doses, indicate that patients with AA value increases in the probabilities of scalp, eyebrow, and eyelash hair regrowth, with the average patient accepting increases in potential treatment‐related risks for the 50‐mg dose in exchange for higher efficacy than 30 mg. The DCE approach to measuring risk tolerance, combined with comparisons to expected benefit and risk differences, can be used to optimize AA treatment dose selection.

## 1 INTRODUCTION

Alopecia areata (AA) is an autoimmune disease characterized by nonscarring hair loss.^1^ Recent evidence demonstrates that treatment with Janus kinase (JAK) inhibitors can lead to substantial hair regrowth in patients with AA, which can significantly improve quality of life (QoL).^2^ Baricitinib, a JAK1/2 inhibitor,^3^ is approved to treat adults with severe AA in the United States, Japan, European Union (EU), China, and several other countries.

Ritlecitinib is an oral inhibitor of JAK3 and tyrosine kinase expressed in hepatocellular carcinoma (TEC) family kinases.^4^ In the ALLEGRO phase 2b/3 (NCT03732807) clinical trial, ritlecitinib demonstrated efficacy and safety in patients with AA at doses of 50 mg and 30 mg once daily (with or without a 200‐mg once‐daily 4‐week loading dose).^5^ Ritlecitinib 50 mg once daily is approved to treat adults and adolescents 12 years and older with severe AA in the United States, EU, Japan, China, and several other countries.^4^

JAK1/2 inhibitors are associated with known risks (e.g., serious infections, malignancies, and thromboembolic events).^6^, ^7^, ^8^, ^9^ It is unknown whether selective JAK3/TEC kinase family inhibition may result in similar risks. In a recent long‐term safety study, ritlecitinib 50 mg (with or without a 4‐week 200‐mg loading dose) was found to be well tolerated in patients with AA.^10^

We conducted a discrete choice experiment (DCE) to quantify the tradeoffs patients were willing to make between AA treatment benefits and potential risks to support ritlecitinib dose selection.^11^ For a 20% increase in the probability of complete or near‐complete scalp hair regrowth, adults with AA were willing to accept substantial 3‐year risks of serious infection, cancer, and blood clots. In a quantitative benefit–risk assessment, ritlecitinib 50 mg had a positive benefit–risk profile compared with placebo.^12^

This study aimed to compare benefit–risk profiles of two doses of ritlecitinib (50 mg vs 30 mg), integrating patient preference and clinical efficacy estimates for ritlecitinib in AA.

## 2 METHODS

### 2.1 Study design

A DCE as previously described^11^ elicited patient preferences for benefit and safety attributes of systemic AA treatments (Table 1). Briefly, respondents completed a series of choice tasks, each consisting of two hypothetical treatment options with variable levels of 3‐year risk probabilities and a no‐treatment option.

**TABLE 1:** DCE attributes.

Attribute	Patient‐facing description	Corresponding clinical trial endpoint
Hair on most or all your scalp	The chance of getting most or all your scalp hair (80% to 100% of your scalp hair) after 24 weeks on treatment	Absolute SALT score of ≤20 (indicating hair regrowth on ≥80% of the scalp) at week 24
Eyebrows	The chance of getting moderate (mildly decreased density and/or short gaps in the eyebrows) or normal eyebrows after 24 weeks on treatment	Two or higher–grade improvement from baseline or absolute score of 3 in the EBA among those with an abnormal baseline score^a^ at week 24
Eyelashes	The chance of getting moderate (mildly decreased density and/or short gaps in the eyelashes) or normal eyelashes after 24 weeks on treatment	Two or higher–grade improvement from baseline or absolute score of 3 in the ELA among those with an abnormal baseline score^b^ at week 24
Risk of serious infections during 3 years of treatment	A serious infection means that you may have to stay in hospital for treatment of the infection and/or receive treatment through an injection. The serious infection may potentially be life‐threatening. You may need to temporarily (until the infection has cleared) or permanently stop your treatment for AA. Examples of such infections may include lung infection, shingles, and urinary tract infection	An infection that results in death, is life‐threatening (immediate risk of death), requires inpatient hospitalization or prolongation of existing hospitalization, results in persistent or significant disability/incapacity (substantial disruption of the ability to conduct normal life functions), or is an important medical event based on investigator judgment^10^
Risk of cancer during 3 years of treatment	Cancer typically requires chemotherapy or surgery, and some cancers can be life‐threatening. Some cancers can be treated or cured with treatment while others may not be treatable. You may need to temporarily or permanently stop your treatment for AA	Any cancer, including malignancies (excluding NMSC), and NMSC
Risk of blood clots during 3 years of treatment	Blood clots require treatment with blood thinning medication, may require you to stay in hospital for treatment, and in some cases may potentially be life‐threatening. You may need to temporarily or permanently stop your treatment for AA	Any thromboembolic event

Abbreviations: AA, alopecia areata; DCE, discrete choice experiment; EBA, Eyebrow Assessment; ELA, Eyelash Assessment; NMSC, nonmelanoma skin cancer; SALT, Severity of Alopecia Tool.

^a^ The EBA is a four‐point scale where 0 = no eyebrow hair, 1 = minimal eyebrow hair, 2 = moderate eyebrow hair, and 3 = normal eyebrow hair.

^b^ The ELA is a four‐point scale where 0 = no eyelash hair, 1 = minimal eyelash hair, 2 = moderate eyelash hair, and 3 = normal eyelash hair.

Benefits included probabilities of achieving ≥80% scalp hair coverage (Severity of Alopecia Tool [SALT] score ≤ 20), moderate to normal eyebrows (two‐unit improvement in Eyebrow Assessment [EBA]), and moderate to normal eyelashes (two‐unit improvement in Eyelash Assessment [ELA]). Potential risks included serious infection, malignancies (including nonmelanoma skin cancer [NMSC]), and blood clots.

### 2.2 Statistical analysis

Preference estimates were used to calculate the maximum acceptable risk (MAR) that patients would accept for expected increases in benefit from choosing a higher dose of ritlecitinib over a lower dose. The MAR for a given 3‐year risk is the percentage‐point increase in that individual risk that yields a utility loss that exactly offsets the utility gain from the increased benefit achieved by switching from ritlecitinib 30 mg to 50 mg, assuming all other risks remain unchanged. MARs were calculated separately for each risk and jointly for all possible risk combinations. Ritlecitinib efficacy benefits for both the 50‐mg and 30‐mg doses were based on clinical data from the ALLEGRO‐2b/3 clinical trial,^5^ using the endpoints in Table 1. Risks were estimated from incidence rates (IRs) per 100 person‐years from an integrated–safety analysis of four clinical studies (two ongoing with a datacut of May 30, 2022); an all‐exposure pool included patients with AA who received ritlecitinib 50 mg once daily with or without an initial once‐daily 4‐week 200‐mg loading dose.

## 3 RESULTS

The preference survey was administered to adult patients (*n* = 201) with physician‐confirmed ≥50% scalp hair loss due to AA in the United States (*n* = 62) and the EU (*n* = 139).^11^ As previously reported, the median age of adult respondents was 39 years (interquartile range [IQR], 29–52 years).^11^ A total of 111 (55%) respondents had an AA diagnosis for >5 years, and 188 (94%) respondents reported moderate to complete scalp hair loss.

In the ALLEGRO‐2b/3 study, the observed efficacy benefit of ritlecitinib 50 mg exceeded the efficacy benefit of 30 mg. At week 24, 23% of participants who received 50 mg achieved response (SALT score ≤ 20), compared with 14.3% of participants who received 30 mg (Table S1).^5^ Similarly, a greater proportion of patients who received 50 mg achieved EBA or ELA responses at week 24 compared with those who received 30 mg (EBA response: 29.0% vs 16.7%; ELA response: 28.9% vs 26.1%).

In exchange for the expected benefits of increasing ritlecitinib dose from 30 mg to 50 mg, respondents were willing to accept increases in risk (Table 2). On average, US patients appeared less tolerant of 3‐year blood clot risks and 3‐year serious infection risks than patients in the EU. Risk tolerance for the 3‐year cancer risk was similar between cohorts.

**TABLE 2:** MAR in exchange for the increase in efficacy benefit achieved by increasing ritlecitinib dose from 30 mg to 50 mg once daily.

3‐year risk	Overall sample MAR, % (95% CI)	US sample MAR, % (95% CI)	EU sample MAR, % (95% CI)
Serious infection	3.88 (3.04–5.14)	2.80 (2.03–3.95)	7.29 (4.89–13.06)
Cancer	1.63 (1.23–2.25)	1.60 (1.04–2.77)	1.71 (1.28–2.46)
Blood clot	5.30 (4.04–7.52)	4.26 (2.97–6.79)	7.61 (5.23–13.87)

Abbreviations: CI, Confidence Interval; EU, European Union; MAR, maximum acceptable risk; US, United States.

**FIGURE 1:**
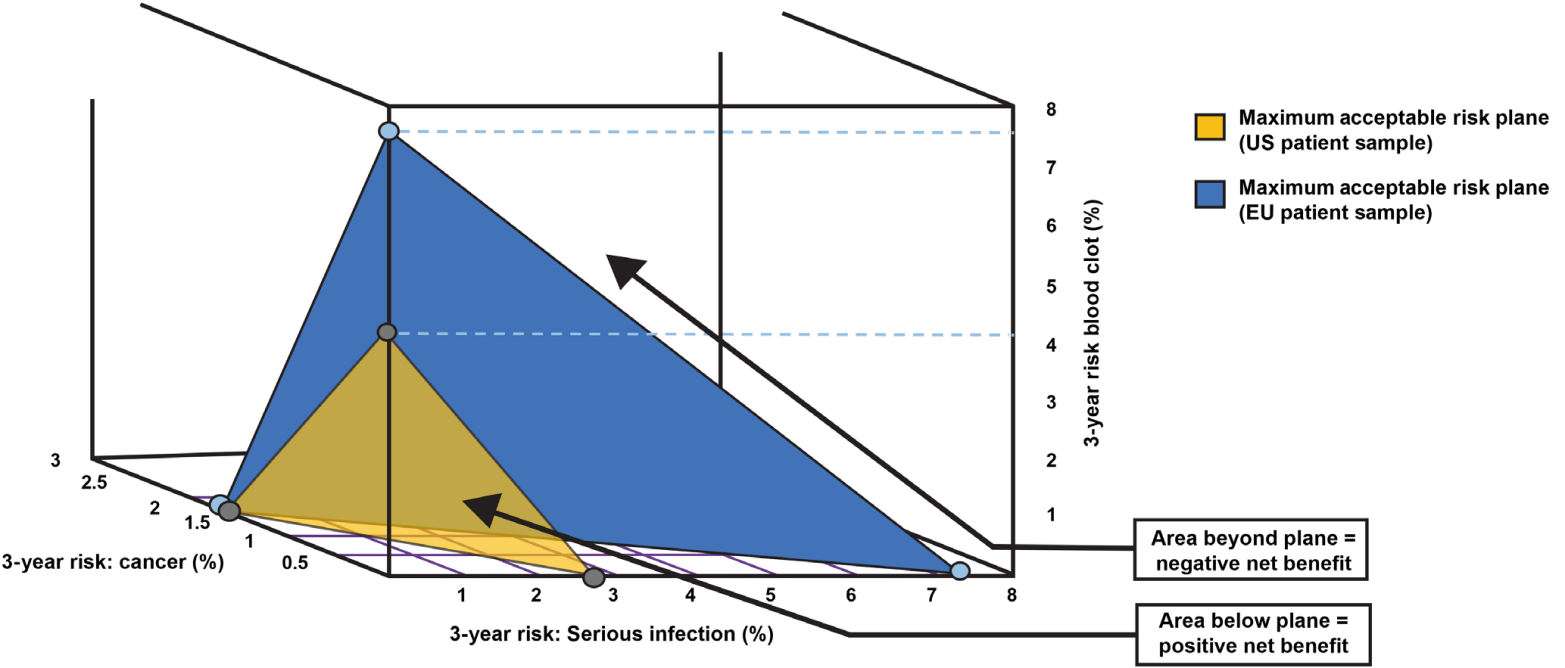
Maximum acceptable combinations of three risks in exchange for the increase in efficacy benefits by switching from ritlecitinib 30 mg to 50 mg once daily for US patients (yellow surface) and European Union (EU) patients (blue surface). Gray dots indicate the maximum acceptable risk (MAR) of each individual risk (assuming no other risk) for the US patient sample. Light blue dots indicate the MAR of each individual risk (assuming no other risk) for the EU patient sample. The dashed lines relate the MAR for the 3‐year risk of blood clot to the corresponding dot in the figure because the risk levels are indicated on a different axis than the corresponding dot.

The combinations of these three risks (i.e., a simultaneous increase in all three) acceptable to patients in the EU and the United States to experience the additional benefit of ritlecitinib 50 mg versus 30 mg are shown in Figure 1. Because the marginal disutility of each percentage‐point increase in risk is constant in the model, the marginal rate of substitution between two risks is constant, and can be represented as a straight line, and the plane representing all combinations of increases in the three risks that will exactly offset the benefit of 50 mg over 30 mg is a flat surface. For example, for each 1‐percentage point reduction in the 3‐year blood clot risk, patients in the overall sample would be willing to accept a 0.73‐percentage point increase in serious infection risk. Likewise, for each 1‐percentage point reduction in 3‐year serious infection risk, patients in the overall sample would be willing to accept *both* a 0.21‐ and 0.68‐percentage point increase in the 3‐year risks of cancer and blood clots, respectively.

IRs for serious infection, cancer (including NMSC), and thromboembolic events from the integrated–safety analysis were 0.66 (95% CI, 0.35–1.13), 0.52 (95% CI, 0.26–0.94), and 0.06 (95% CI, 0–0.29), respectively.^10^ Multiplying the IRs by three to approximate 3‐year risks, and applying the strong assumption that IRs are proportional to the dose (i.e., IR for 30 mg = 0.6× IR for 50 mg), the difference in risks between the 30‐mg and 50‐mg doses would be approximately 1.18%, 0.94%, and 0.001% for serious infection, cancer, and thromboembolic events, respectively. Each rate was less than the corresponding MAR estimate, and this conclusion holds in multiple scenarios with different assumptions about the risk difference between doses.

## 4 DISCUSSION

EU‐ and less risk‐tolerant US‐patient samples accepted a relatively high level of any combination of 3‐year risks in exchange for the increase in benefit of ritlecitinib 50 mg over 30 mg, and this exceeded the difference in risk between the two doses in multiple scenario analyses. Therefore, the incremental net benefit of the 50‐mg dose compared with the 30‐mg dose was positive.

These results are not surprising given that AA‐associated hair loss has a major impact on patient identity and mental health. AA is associated with reduced QoL and a large psychosocial burden; patients often report stigma in their daily lives.^13^, ^14^, ^15^ Because of this burden, patients place high value on treatments that can induce hair growth and are willing to accept serious potential risks in exchange.^11^, ^12^ The absolute percent increase in risk that patients were willing to tolerate in exchange for the increase in benefit associated with increasing ritlecitinib dose from 30 mg to 50 mg was greater than any estimated increase in actual 3‐year risk between the two doses. Specifically, adult patients with AA were willing to accept increases in safety risks of serious infection, cancer, and thromboembolism with a higher dose of ritlecitinib (50 mg over 30 mg) for increased probabilities of scalp, eyebrow, and eyelash hair regrowth. This study demonstrates that patient preference data can inform optimal treatment dose selection to support patient‐centric medicinal product development decisions, regulatory benefit–risk assessment, and physician and patient shared decision‐making.

## CONFLICT OF INTEREST STATEMENT

BH, JM, EL, EW, DW, and RW are employees of and may hold stock/stock options in Pfizer Inc. At the time of the study, SHZ was an employee of and may hold stock/stock options in Pfizer, Inc. CW, MT, and NK are employees of Evidera, which received funding from Pfizer to conduct this study. At the time of the study, TT was an employee of Evidera.

## Supporting information


Table S1.

## Data Availability

Upon request, and subject to review, Pfizer will provide the data that support the findings of this study. Subject to certain criteria, conditions, and exceptions, Pfizer may also provide access to the related individual deidentified participant data. See https://www.pfizer.com/science/clinical‐trials/trial‐data‐and‐results for more information.
