# Supplementary material for: Using patient preference to inform ritlecitinib dose selection for alopecia areata treatment
Source: J Dermatol. 2025 Jan 15;52(3):510–4. doi: 10.1111/1346-8138.17628 (PMC11883726; doi:10.1111/1346-8138.17628)
Supplement: Supplementary file 1 — Table S1. [file JDE-52-510-s001.docx]

**Using Patient Preference to Inform Ritlecitinib Dose Selection for Alopecia Areata Treatment**

Brett Hauber^1^, Chiara Whichello^2^, Jonathan Mauer^1^, Ernest Law^1^, Myrto Trapali^2^, Edward Whalen^1^, Dalia Wajsbrot^1^, Nicolas Krucien^2^, Tommi Tervonen^3*^, Samuel H Zwillich^4*^, Robert Wolk^4^

^1^Pfizer Inc., New York, NY, USA; ^2^Evidera, London, UK; ^3^Evidera, Zurich, Switzerland; ^4^Pfizer, Groton, CT, USA.

*Affiliation at the time of the study

**SUPPORTING INFORMATION**

**Table S1.** Ritlecitinib effects in ALLEGRO phase 2b/3 clinical trial^5^

| **Benefit** | **Ritlecitinib 50-mg QD** | **Ritlecitinib 30-mg QD** |
| --- | --- | --- |
| **Response based on SALT score ≤20 at Week 24, n/N (%)** | 29/124 (23.4) | 17/119 (14.3) |
| **EBA response^a^ at Week 24, n/N (%)** | 29/100 (29.0) | 17/102 (16.7) |
| **ELA response^b^ at Week 24 n/N (%)** | 26/90 (28.9) | 24/92 (26.1) |

EBA, eyebrow assessment; ELA, eyelash assessment; QD, once daily; SALT, Severity of Alopecia Tool.

^a^ Response defined as ≥2-grade improvement from baseline or absolute score of 3 in the EBA among those with an abnormal baseline score.

^b^ Response defined as ≥2-grade improvement from baseline or absolute score of 3 in the ELA among those with an abnormal baseline score.
